# A New World of Biomarkers and Therapeutics for Female Reproductive System and Breast Cancers: Circular RNAs

**DOI:** 10.3389/fcell.2020.00050

**Published:** 2020-03-09

**Authors:** Anh M. Tran, Ghanbar Mahmoodi Chalbatani, Lea Berland, Mireia Cruz De los Santos, Priyank Raj, Seyed Amir Jalali, Elahe Gharagouzloo, Cristina Ivan, Mihnea P. Dragomir, George A. Calin

**Affiliations:** ^1^Department of Experimental Therapeutics, The University of Texas MD Anderson Cancer Center, Houston, TX, United States; ^2^Cancer Research Center, Cancer Institute of Iran, Tehran University of Medical Sciences, Tehran, Iran; ^3^Department of Medical Immunology, School of Medicine, Tehran University of Medical Sciences, Tehran, Iran; ^4^Department of Immunology, School of Medicine, Shahid Beheshti University of Medical Sciences, Tehran, Iran; ^5^Center for RNA Interference and Non-Coding RNAs, The University of Texas MD Anderson Cancer Center, Houston, TX, United States; ^6^Department of Surgery, Fundeni Clinical Hospital, Carol Davila University of Medicine and Pharmacy, Bucharest, Romania

**Keywords:** circular RNAs, cancer, cancer therapy, gynecological cancer, breast cancer, female reproductive system

## Abstract

As one of the most recently (re)discovered types of non-coding RNAs (ncRNA), circular RNAs (circRNAs) differentiate from other ncRNAs by a specific biogenesis, high stability, and distinct functions. The biogenesis of circRNAs can be categorized into three mechanisms that permit the back-splicing reaction: exon-skipping, pairing of neighboring introns, and dimerization of RNA-binding proteins. Regarding their stability, circRNAs have no free ends, specific to linear RNA molecules, prompting a longer half-life and resistance to exonuclease-mediated activity by RNase R, bypassing the common RNA turnover process. Regarding their functions, circular transcripts can be categorized into four broad roles: miRNA sponging, protein binding, regulation of transcription, and coding for proteins and peptides. Female reproductive system (including mainly ovarian, corpus, and cervix uteri cancers) and breast cancers are the primary causes of death in women worldwide, accounting for over 1,212,772 deaths in 2018. We consider that a better understanding of the molecular pathophysiology through the study of coding and non-coding RNA regulators could improve the diagnosis and therapeutics of these cancers. Developments in the field of circRNA in regard to breast or gynecological cancers are recent, with most circRNA-related discoveries having been made in the last 2 years. Therefore, in this review we summarize the newly detected roles of circRNAs in female reproductive system (cervical cancer, ovarian cancer, and endometrial cancer) and breast cancers. We argue that circRNAs can become essential elements of the diagnostic and therapeutic tools for female reproductive system cancers in the future.

## Introduction

Contrary to the “central dogma of biology” described by Francis Crick (1970) in which information passes from DNA to RNA and finally to protein, non-coding RNAs (ncRNAs) usually do not participate in protein synthesis (Fabbri et al., 2008, 2019; Bayraktar et al., 2017; Dragomir et al., 2018). Despite not carrying any coding sequences, ncRNAs are well studied across multiple disease disciplines and are sub-classified as microRNAs (miRNAs), transcribed pyknons, small nucleolar RNAs, PIWI-interacting RNAs, long non-coding RNAs, and circular RNAs (circRNAs) (Maxwell and Fournier, 1995; Siomi et al., 2011; Spizzo et al., 2012; Bartel, 2018; Zhang Z. et al., 2018; Dragomir et al., 2019). Among these diverse sub-classes of ncRNAs, circRNA transcripts are the newest addition, recently emerging as a novel class of endogenous RNAs that exist ubiquitously in mammalian cells (Bolha et al., 2017; Dragomir and Calin, 2018a). CircRNAs were primarily detected as viruses in 1970 by using electron microscopy; in 1979 researchers found them to exist in eukaryotic cells (Sanger et al., 1976; Hsu and Coca-Prados, 1979; Chen L. et al., 2015). In 2012, Salzman et al. found abundant circRNA transcripts from different human genes, showing that exons scramble in a non-canonical order and stabilize in a circular conformation (Salzman et al., 2012). Today, liquid biopsies for clinical trials are conducted based on the stable existence of circRNAs in human tissues and fluids: serum and urine (Esteller, 2011).

As one of the most recently discovered ones in the ncRNA world, circRNAs differentiate from other ncRNAs by a specific biogenesis, high stability, and functions. Generally, the biogenesis of circRNAs can be categorized into three distinct mechanisms that permit the back-splicing reaction: exon-skipping, pairing of neighboring introns, and dimerization of RNA binding proteins (the last two being direct back-splicing biogenesis mechanisms) (Li et al., 2018). These varied mechanisms lead to exonic, intronic, and exon-intron circRNAs. Present in a circular form with no free ends specific for linear RNA molecules, circRNAs are more resistant to the enzymatic activity of RNase R, bypassing common RNA turnover process (Suzuki and Tsukahara, 2014). The advantage of this longer life span compared to their messenger RNA (mRNA) counterparts makes circRNAs attractive diagnostic and therapeutic tools in the future. The specific characteristics of circRNA have already been exploited in developing biomarkers for the diagnosis and screening of different pathologies such as atherosclerosis, prion disease, neurological disorders, and human cancers (Braicu et al., 2019).

Recent studies outlined several important roles of circRNAs in different molecular biology pathways: miRNA sponges, regulators of RNA binding protein (RBP), regulators of transcription, and coding for proteins and peptides (Zheng Q.P. et al., 2016; Peng et al., 2017; Zang et al., 2018). Because of their tremendous activities in important genetic pathways, especially oncology, scientists can envision two different therapeutic potentials of circRNAs: inhibiting the circRNAs that are carcinogenic and overexpressed in tumor tissues or restoring circRNAs with tumor suppressor functions that are down-regulated in the tumor (Dragomir and Calin, 2018b). There are many more mechanisms and functions of this new class of transcript yet to be learned, however, with the vast development of computational strategies, the kernels of circRNAs in cancer biology are being discovered.

## The Biogenesis of circRNAs

CircRNAs are usually generated from pre-mRNAs, process-facilitated by RNA Polymerase II (Pol II) (Chen, 2016). What makes circRNAs so special is the covalent closed loop, without poly(A) tails at 3′ end, that usually decides the fate of many RNA transcripts (Jeck and Sharpless, 2014). Interestingly, a high degree of conservation in circRNAs gene expression is found across eukaryotic species (Memczak et al., 2013; Wang et al., 2014). Although most of them are not well expressed, there are multiple circRNAs more abundantly present than their linear mRNA analogs. The formation of circRNAs stems from intronic, exonic, and intergenic regions, or even 5′ and 3′ untranslated segments (Memczak et al., 2013; Zhang et al., 2013; Lei et al., 2018). In general, we categorize circRNAs into three types: exonic, intronic, and exon-intron circRNAs, based on their distinct composition and circularization mechanism (Xu S. et al., 2018). The splicing regulatory mechanisms of circRNA biogenesis are diverse from the linear isoforms. Although there remain unanswered clarifications about circRNA biogenesis, we define the main process as back-splicing (Nigro et al., 1991). CircRNAs display distinct and diverse back-splicing events under catalysis of the canonical spliceosomal mechanism across different cell lines (Ashwal-Fluss et al., 2014; Starke et al., 2015; Wang and Wang, 2015; Zhang et al., 2016). Three models have been proposed to specify each mechanism of circRNA formation: exon-skipping, intron pairing, and RNA-binding protein interactions (Vicens and Westhof, 2014; Barrett et al., 2015; Dong et al., 2017).

**FIGURE 1:**
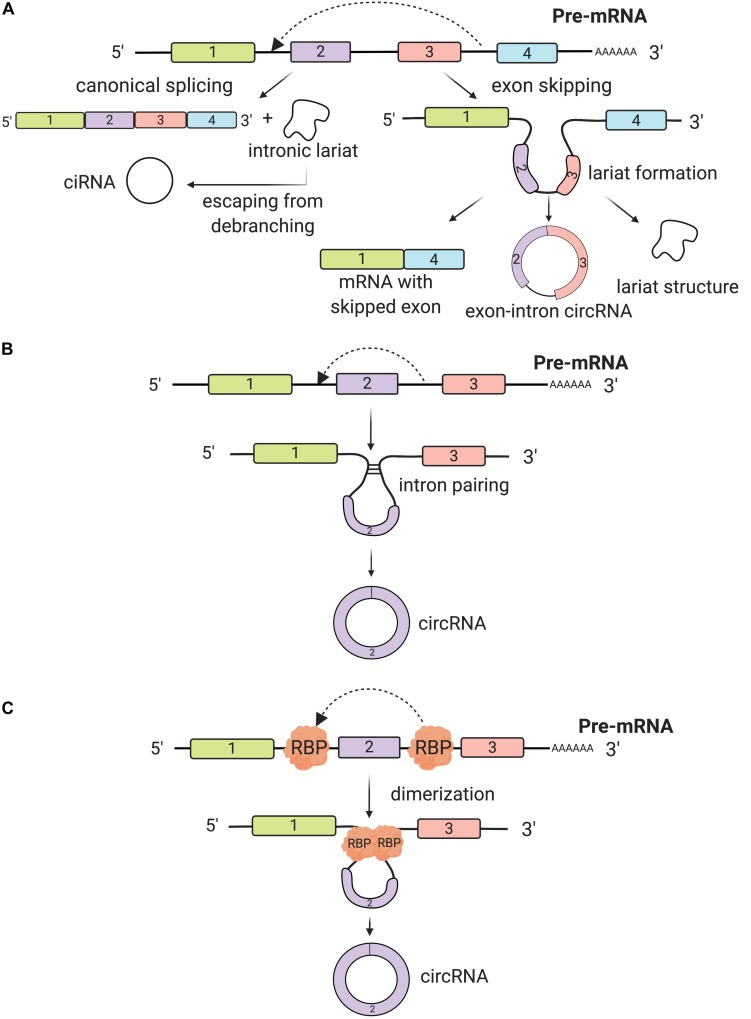
Biogenesis of circRNAs. **(A)** The back-splicing process can take place because of exon skipping mechanism, which leads to lariat formation. This process is a non-canonical splicing pathway and three different products are synthesized: a circRNA, a mRNA with skipped exons and a lariat structure. Additionally, intron lariats, by escaping from debranching, can form intronic circRNAs (ciRNAs). **(B)** Back-splicing can be induced by intron pairing (often Alu repeats). Introns covalently bind together and a circRNA is synthesized. **(C)** RNA binding proteins (RBP) bind usually introns flanking the exon(s) that will form the circRNA. RBP dimerize promoting the back-splicing process.

The first model that can give rise to back-splicing is exon skipping, in which one or multiple exons of the mature mRNA will be missing. In this model, the lariat-driven circularization proceeds as two non-adjacent exons join together, finally producing a mRNA with skipped exons, a circular RNA transcript and a lariat structure. Additionally, intronic lariats can form intronic circRNAs (ciRNAs) if these circular loops escape from the activity of debranching enzyme (DBR1 debranching RNA lariats 1). The existence of ciRNAs depends on a 7-nt GU-rich motif, located in the proximity of the 5′ splice site and a 11-nt C-rich motif close to the branchpoint (Zhang et al., 2013; Kristensen et al., 2019; Figure 1A). The relationship between the circRNA and exon skipping transcription has been demonstrated by Jeck et al.; his group has characterized non-colinear exons of more than 25,000 different RNA species in human fibroblasts via high-throughput sequencing that follows this mechanism (Jeck et al., 2013; Barrett et al., 2015).

The second biogenesis mechanism of circRNAs is intron pairing-driven circularization. In this biogenesis mechanism, two introns flanking the exon/exons of a pre-mRNA have a structure capable of joining each other. The flanking introns approach each other creating a secondary conformation that makes the splice sites possible to carry on back-splicing (Figure 1B). Most of the intron-pairing patterns are promoted by ALU repeats. By using the bioinformatics database from UCSC, Ivanov et al. predicted human circRNAs genome-wide, based on the sequences correlated with these ALU consistent repeats (Ivanov et al., 2015). In a follow-up study, Zhang et al. (2014) found that the length of flanking introns does not necessarily control the biogenesis of circRNAs. However, the more extended length of introns, the more chances for them to have more ALU elements, consequently enhancing exon circularization (Zhang et al., 2014). Adenosine deaminase 1 acting on RNA (ADAR1) is involved in the intron-pairing process of circRNA formation. Known to interact with ALU repeats, ADAR can decrease the pairing activity of ALU repeats, which prevents the formation of circRNAs (Athanasiadis et al., 2004). In another study, ADAR1 was proven to be a double-stranded RNA (dsRNA) binding protein that interrupts miRNA processing (Chen T. et al., 2015). Therefore, there is a possibility that this protein factor regulates circRNA formation through a direct mechanism of dsRNA binding activity.

Thirdly, another circRNA formation mechanism is by RNA-binding proteins (RBPs). This mechanism involves protein factors that are able to bind to pre-mRNAs to connect the flanking introns together. This process is induced by protein dimerization, which creates an RNA loop. One of the most popular RBPs responsible for circRNA biogenesis is muscleblind like splicing regulator 1 (MBNL1) protein (Chen and Yang, 2015). CircRNA MBL/MBNL1 contains conserved MBL binding sites on its own so that it is easily bound to MBL protein (Ashwal-Fluss et al., 2014; Du et al., 2017; Taylor et al., 2018). This binding interaction promotes circMBL biosynthesis, and the MBL levels are crucial in determining the circularization rates of bracketed exons. MBNL1 proteins tie to neighboring introns of their own pre-mRNA and by dimerizing they link the introns together and prompt circularization (Figure 1C). There are also other important RBPs controlling circRNA biogenesis, such as nuclear factor 90 and 110 (NF90/NF110) (Li et al., 2017), QKI (Conn et al., 2015), and FUS (Errichelli et al., 2017), all of which promote the back-splicing process.

Over the past decade, extensive research on circRNA biogenesis with various proposed mechanisms was carried out. Although circRNAs were first believed to be just transcriptional noise from the RNA splicing process, more and more discoveries have confirmed that they have a strictly regulated biosynthesis. However, the biogenesis is not yet fully characterized, which opens broader space for researchers to carry out further investigations. More research is needed in the future to decipher the multiple aspects of circRNA biogenesis so that we can better categorize and detect them via computational genomic strategies. The reasons and mechanism behind how different circRNAs are formed are also vital in studying their relationship with other gene targets for different diseases such as cancer.

## Functions of circRNAs

A general and in-depth overview of the characteristics and functions of circRNAs is still lacking. However, the high degree of conservation between different species may suggest some important roles in the physiological cellular mechanisms (Wang et al., 2014). CircRNAs’ transcriptional expression is cell-specific and differentially detected between healthy and disease samples, which turns them into a potential candidate of illness-related biomarkers (Salzman et al., 2013). Also, circRNAs’ functionality has been suggested by their long half-life compared to other RNA counterparts. Endogenous circRNAs lack the 5′ and 3′ ends due to circularization, so they can escape from the exonuclease-mediated degradation, being actively resistant to multiple RNA turnover mechanisms (Enuka et al., 2016; Kaczmarek et al., 2017). Additionally, for some circRNAs, complex *in vivo* studies were performed and proved their functionality. Recently, a CDR1 as knockout mouse model was developed and showed that this circRNA binds miR-7 and miR-671 and deregulates their expression *in vivo*, leading to impaired brain function (Piwecka et al., 2017). Such *in vivo* models are highly necessary to understand the complex functions of circRNAs in female reproductive system and breast cancers.

With all of these arguments, scientists are more objective that circRNAs actually carry out important roles in regulating different molecular pathways by four possible functions: miRNA sponging, protein binding, direct/indirect regulation of transcription, or coding for proteins and peptides.

### miRNA Sponging

**FIGURE 2:**
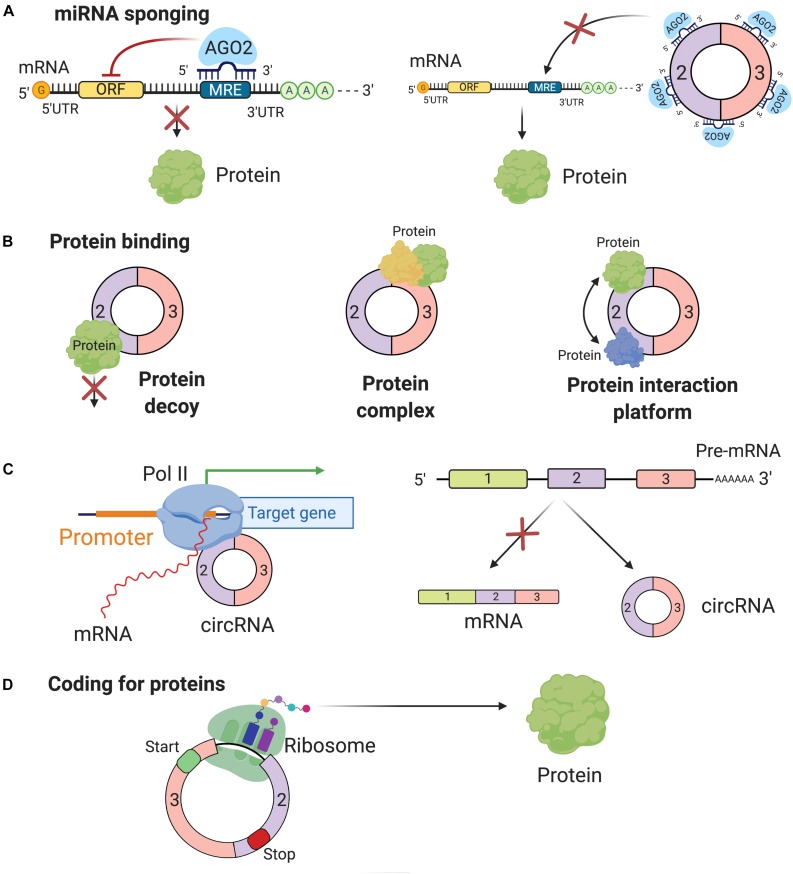
Function of circRNAs. **(A)** The most common function of circRNAs is miRNA sponging. By sponging miRNAs, circRNAs inhibit miRNA capacity to perform their post-transcriptional inhibition. **(B)** By binding proteins circRNAs can block their function (protein decoy), can build protein complexes, which include multiple proteins and have complex regulatory functions or are scaffolds for protein-protein interactions. **(C)** CircRNAs can affect the biogenesis of other genes by directly interacting with the promoter region at the DNA level or simply the preferential biogenesis of circRNAs inhibits the formation of functional mRNAs. **(D)** Recently, it was reported that some circRNAs have coding potential and are translated into proteins.

One of the most studied functions of ncRNAs is miRNA sponging, defined as the anti-sense partial complementarity interaction between a ncRNA (other than a miRNA) and a miRNA. Belonging to the small ncRNAs class, miRNA down-regulates gene expression at mRNA level (Calin and Croce, 2006; Friedman et al., 2009; Fabbri and Calin, 2010; Almeida et al., 2012; Dragomir et al., 2018). Mathematical modeling indicated that the miRNA sponging depends on the intracellular mobility mechanism of miRNAs, which is characterized to be of intermittent active transport type (Vasilescu et al., 2016). The miRNA–circRNA interaction was brought into attention due to its complex cascade of gene expression regulation. One of the first discoveries on circRNA as miRNA sponging was from Hanssen’s lab, when they found more than 70 conserved miRNA interaction sites (for the same miRNA) on ciRS-7 (CDR1as) (Hansen et al., 2013). Because of their specific structure, circRNAs can stay away from miRNAs’ destabilization and degradation via miRNA-mediated deadenylation (Hansen et al., 2011). CircRNAs can block the binding of miRNA base-pairing to its target mRNA. By sequestering the negative regulatory activities of miRNAs on mRNAs, circRNAs indirectly affect the expression level of these translational brake (Figure 2A). Salmena et al. (2011) and Tay et al. (2014) also suggested mRNAs and circRNAs compete with each other for binding the same miRNAs, via miRNA response elements (MREs).

Despite being considered a classical model of circRNA functions, scientists still raise many arguments regarding miRNA sponging as a general function of all circRNAs. Some recent studies lean toward a controversial view, that some circRNAs cannot always act as a miRNA sequester. For example, Militello et al., using computational algorithms, showed that only two circRNAs out of 7112 human circRNAs have more predicted miRNA-binding site than expected by chance (Militello et al., 2017). However, more and more concrete evidence on individual circRNAs sponging miRNAs are validated in several cancer models. There are up to 822 studies available on PubMed based on the search terms “circRNAs,” “miRNA sponge,” and “cancer” accessed by July 2019. Hence, in this review, we mostly analyze the roles of circRNAs in gynecological and breast cancers via miRNA sponging mechanism.

### Protein Binding

In addition to sponging miRNAs, circRNAs also bind to different RBPs and have different potential roles: inhibiting the function of proteins (protein decoys), facilitating the formation of protein complexes, and permitting the interaction between different proteins (interaction platform) (Zang et al., 2018; Figure 2B). Several well-known circRNAs associate with RBPs; the best-known examples are circ-MBL, circ-Foxo3, and hsa_circ_0000020 (Ashwal-Fluss et al., 2014; Du et al., 2016; Dudekula et al., 2016).

Ashwal-Fluss demonstrated that the splicing factor muscleblind (MBL/MBNL1) circRNA (circ-MBL) and its neighboring introns have conserved MBL interaction sites. Specifically, circRNAs can get tuned in their biosynthesis, depending on the level of MBL proteins. There is convincing evidence showing that circRNAs production is co-transcriptional and competes with the canonical pre-mRNA splicing, suggesting its potential role in gene regulation (Ashwal-Fluss et al., 2014).

Circ-Foxo3 is another circRNA that has a capability of interacting with different proteins related to cell progression. Two common target proteins of circ-Foxo3 are cyclin-dependent kinase inhibitor 1 (or p21) and cyclin-dependent kinase 2 (CDK2). CDK2 coordinates the activities of G1/S and S/G2 changeover in the cell cycle (Peng et al., 2016). In contrast, p21 retards the cell cycle progression by restraining various interactions of cyclin A and cyclin E (Karimian et al., 2016). Circ-Foxo3 forms a complex together with these two proteins, which facilitates the communication between p21 and CDK2, inhibiting the normal function of the latter (Du et al., 2016).

Using computational methods, Dudekula et al. (2016) confirmed the footprints of flanking sequences of hsa_circ_0000020 on binding to some RBPs, including HuR, FMRP, and ElF4A3 at a very high frequency and hypothesized that this circRNA may act as a protein decoy. More than 117,000 circRNAs were found to bind with eukaryotic translation initiation factor 4A3 (ElF4A3). However, most of the uncertainties are based on how this binding between circRNAs and RBPs contribute to RBP-related functions.

### Regulation of Transcription

CircRNAs also play a role in transcription regulation, enhancing transcription at the transcriptional level. Focusing on transcriptional level, circRNAs have two regulatory paths: one at the initiation step and one at the elongation step. During the initiation step, the role played by circRNAs involves the formation of the pre-initiation complex. Li et al. (2015) found two exon-intron circRNAs (EIciRNAs) exclusively localized in the nucleus, and the knockdown assay of these circular transcripts results in decreased levels of their parental genes. Subsequent experiments revealed a particular collaboration *in cis* between the EIciRNAs and U1 snRNA, which forms a complex together with Pol II at the DNA level at the site of the promoter. During the elongation phase, the interaction of Elongating Polymerase II with ciRNAs was described. CiRNAs accumulate at their site of transcription and increase parental genes transcription elongation by interacting with RNA polymerase II (Zhang et al., 2013). These features distinguish them from circRNAs, localized in the cytoplasm, functioning mainly as miRNA sponges. Additionally, the biogenesis of circRNAs via exon skipping can be seen as a passive function of the circular transcripts. The production of a circRNA also leads to the synthesis of a mRNA, which misses one or multiple exons and most likely alters its coding capacity (Dragomir and Calin, 2018a; Figure 2C).

### Coding for Proteins or Peptides

In general, the translation of mRNAs into proteins begins with the recognition in 5′ UTR of the initiation codon and ends with in the 3′ region the stop codon (Hershey et al., 2012). Since circRNAs do not have this initiation codon, it was long thought that they could not be translated. Most probably, circRNAs do not need large polyribosomes, but just a limited number ribosomes that are sufficient to translate the circRNAs into peptides and proteins (Panda et al., 2017; Figure 2D). Confirming these statements, several studies reported the presence of small peptides, often less than 100 amino acids encoded by supposedly non-coding regions of the genome. Among these “non-coding” regions, some are recognized as circRNAs and contain short ORFs, similar to lncRNA, which can actually generate small proteins or micropeptides (Panda et al., 2017; Yang Y. et al., 2017; Pan et al., 2018). Furthermore, the origin of these circRNAs, mostly exonic, and their cellular compartmentalization, mostly cytoplasmic, add evidence for their translation into functional peptides (Jeck et al., 2013).

## circRNAs and Female Reproductive System and Breast Cancers

Female reproductive system (including cervical cancer (CC), ovarian cancer (OC), and endometrial cancer (EC), as well as more uncommon neoplasia, such as vulvar or vaginal cancers, categorized also as gynecological cancers) and breast cancers (BC) are a leading cause of death worldwide. Their incidence increases continuously and is expected to reach 109,000 women for gynecological cancers and 268,600 for BCs in 2019 in the United States. Mortality is also significant among these patients, although it varies between countries. In the United States, <41,760 women will die from BC and 33,100 from gynecological cancer in 2019 according to the American Cancer Society (Bray et al., 2018).

These cancers have a strong genetic predisposition. Hereditary BC and OC are syndromes, with an autosomal-dominant pattern of transmission, which involves an increased predisposition to OC, BC, or both. BRCA1 and BRCA2 are the genes most often found mutated and increase the risk for early age onset BC and/or OC, and often after a first cancer a second cancer is common (Miki et al., 1994; Wooster et al., 1995; Hartmann and Lindor, 2016). Recent data shows that not only other coding genes (Calin et al., 2005), but also non-coding ones, including circRNAs, play a role in the genetic predisposition for breast and reproductive system cancers (Table 1).

**TABLE 1:** Summary of deregulated circRNAs in breast and female reproductive system cancers.

**Disease**	**circRNA**	**Target**	**Up/Down**	**Function**	**References**
Breast cancer	circFBXW7	miR-197-3p and encodes a tumor suppressor protein, FBXW7-185aa	Down	Tumor Suppressor	Ye et al., 2019a
	circ-ABCB10	miR-1271	Up	Oncogene	Liang et al., 2017
	circ_0103552	miR-1236	Up	Oncogene	Yang L. et al., 2019
	hsa_circ_0004771	miR-653 and indirectly ZEB2	Up	Oncogene	Xie R. et al., 2019
	hsa_circ_0072309	miR-492	Down	Tumor Suppressor	Yan et al., 2019
	hsa_circ_001783	miR-200c-3p and indirectly ZEB1, ZEB2, and ETS1	Up	Oncogene	Liu et al., 2019
	circ_0005230	miR-618 and indirectly CBX8	Up	Oncogene	Xu Y. et al., 2018
	hsa_circ_00052112	miR-125a-5p and indirectly BAP1	Up	Oncogene	Zhang H.D. et al., 2018
	hsa_circ_0007534	miR-593 and indirectly MUC1	Up	Oncogene	Song and Xiao, 2018
	hsa_circ_0001982	miR-143	Up	Oncogene	Tang et al., 2017
	circGFRA1	miR-34a and indirectly GRAF1	Up	Oncogene	He et al., 2017
	circ-Foxo3	miR-22, miR-136^∗^, miR-138, miR-149^∗^, miR-433, miR-762, miR-3614–5p and miR-3622b–5p and indirectly Foxo3	Down	Tumor Suppressor	Yang et al., 2016
	circANKS1B	miR-148a-3p and miR-152-3p and indirectly USF1	Up	Oncogene	Zeng et al., 2018
	circTADA2As	miR-203a-3p and indirectly SOCS3	Down	Tumor Suppressor	Xu et al., 2019
	circAGFG1	miR-195-5p and indirectly CCNE1	Up	Oncogene	Yang R. et al., 2019
Cervical cancer	circAMOTL1	miR-485-5p and indirectly AMOTL1	Up	Oncogene	Ou et al., 2019
	circE7	Encoding the viral oncoprotein E7	Up	Oncogene	Zhao J. et al., 2019
	hsa_circ_0018289	miR-497	Up	Oncogene	Gao et al., 2017
	circEIF4G2	miR-218 and indirectly HOXA1	Up	Oncogene	Mao et al., 2019
	hsa_circRNA_101996	miR-8075 and indirectly TPX2	Up	Oncogene	Song et al., 2019
	hsa_circ_0000263	miR-150-5p and indirectly MDM4	Up	Oncogene	Cai et al., 2019
	hsa_circ_0067934	miR-545 and indirectly EIF3C	Up	Oncogene	Hu et al., 2019
	circRNA8924	miR-518-5p and miR-519-5p	Up	Oncogene	Liu J.M. et al., 2018
	hsa_circ_0023404	miR-136 and indirectly TFCP2	Up	Oncogene	Zhang J.H. et al., 2018
	circRNA-000284	miR-506 and indirectly Snail-2	Up	Oncogene	Ma et al., 2018
	circSMARCA5	miR-620	Down	Tumor Suppressor	Tian and Liang, 2018
	circ-ATP8A2	miR-433 and indirectly EGFR	Up	Oncogene	Ding and Zhang, 2019
Ovarian cancer	CDR1as	miR-1270	Down	Tumor Suppressor	Zhao Z. et al., 2019
	circHIPK3	Not confirmed	Down	Tumor Suppressor	Teng et al., 2019
	circRNA1656	N/A	Down	Tumor Suppressor	Gao et al., 2019
	circ-ITCH	miR-145 and indirectly RASA1	Down	Tumor Suppressor	Hu et al., 2018
	hsa_circ_0061140	miR-370 and indirectly FOXM1	Up	Oncogene	Chen Q.Z. et al., 2018
	circEPSTI1	miR-942 and indirectly EPSTI1	Up	Oncogene	Xie J. et al., 2019
Endometrial cancer	hsa-circ-0039659	hsa-miR-542-3p and hsa-let-7c-5p	Up	Oncogene	Ye et al., 2019b
	circ-ZNF91	miR-23b and miR-199	Up	N/A	Chen B.J. et al., 2018

### Breast Cancer

Breast cancers remains the most frequent form of women’s cancer and ranks second for cancers related to death in women (Bray et al., 2018). Emerging from the cells in the breast secretory system, made of lobules and galactophoric channels, BCs are mainly ductal or lobular adenocarcinomas. The development of targeted anti-HER2 therapies has made a significant improvement in the prognosis of metastatic BC, and the emergence of immunotherapy raises hopes for a better management of these patients (Libson and Lippman, 2014; Nanda et al., 2016). Despite this, BC still remains an important challenge for physicians and scientists in the battle of finding the most efficient diagnosis and treatments for BC patients. Most deaths caused by BC stem from the relapse and metastasis to other distant organs when conventional treatments such as surgery or curative chemotherapy are no longer an option–only 26% of stage 4 patients reach 5 years of survival (Braden et al., 2014; Peart, 2017).

Therefore, it is urgent to investigate the cascade of molecular events leading to breast malignancies, especially before metastasis development, in order to target the tumors as soon as possible. Understanding the mechanistic basis of genetic and epigenetic changes in BC can guide us to develop novel diagnostic and therapeutic strategies for treating this fatal disease. CircRNAs were proved to be involved in different hallmarks of BC such as proliferation, apoptosis, and activating invasion and metastasis (Zhou et al., 2019).

One of the best characterized circRNAs in BC is circFBXW7. Carrying two functions, protein coding and miRNA sponging, circFBXW7 was discovered to be down-regulated and negatively correlate with tumor development and lymph node metastases in triple-negative breast cancer (TNBC). CircFBXW7 is located mainly in the cytoplasm, suggesting its potential relation to miRNA activities. Based on computational analysis, Ye et al. (2019a) have analyzed and confirmed miR-197-3p as one target of circFBXW7. As a miRNA sponge, circFBXW7 decreases the expression level of miR-197-3p and inhibits tumor progression. In 473 TNBC samples, a Spearman correlation analysis provided a positive correlation between circFBXW7 and its host gene FBXW7, which is regulated by miR-197-3p. CircFBXW7 also encodes FBXW7-185aa protein, which up-regulates the tumor suppressor FBXW7 and degrades c-Myc oncogene, further inhibiting TNBC proliferation and migration. These data have strengthened the concept of using circFBXW7 as a potential diagnostic and therapeutic tool for TNBC patients in the future.

By using microarray analysis and RT-qPCR, Liang et al. (2017) conducted a screening on different circRNAs expressed in BC tissues and adjacent non-cancerous tissues. Across a total of 2,587 circRNAs analyzed, circ-ABC10 is overexpressed in cancerous tissues, five to ten times higher than in healthy tissues. Knockdown of circ-ABC10 prevents BC cells from proliferation and initiates apoptosis, suggesting its oncogenic characteristic. Mechanistically, circ-ABC10 was suggested to bind miR-1271 and inhibit its functional activity. The same pattern was observed in circ_0103552, a 920-nucleotide circRNA. Yang L. et al. (2019) detected an up-regulation of its expression in BC cells *in vitro*, and associated its level with higher TNM stages and lymph node invasion in patient samples. The rate of apoptosis increases noticeably in the absence of circ_0103552, while overexpressing this circRNA leads to a boost in cell growth ability in multiple BC cell lines. This study also showed that this circRNA does not act alone, but under a negative association with miR-1236. Furthermore, Xie R. et al. (2019) used high-throughput circRNA sequencing to examine the expression level of hsa_circ_0004771. Not only being up-regulated in BC tissues, this circRNA was proved to target miR-653 and decrease its expression and inhibit its function. They showed that, if not sponged, miR-653 directly binds to 3′-UTR of the mRNA of ZEB2 (Zinc finger E-box binding homeobox 2).

Another circRNA called hsa_circ_0072309 is expressed at abnormally high levels in patients with BC and is linked to poor overall survival (OS) rates (Yan et al., 2019). In order to uncover the mechanism of hsa_circ_0072309, Yan et al. used a luciferase reporter assay to confirm the direct interactions between this circRNA and miR-492. The overexpression and knockdown experiments proved the oncogenic role of hsa_circ_0072309, endorsing the potential of using it as a biomarker for BC diagnosis. Liu et al. (2019) analyzed 923 circRNAs and 100 miRNAs with over 37,000 possible interactions. Among 923 investigated circRNAs, hsa_circ_001783 was established to have the highest rank score among all BC-associated circRNAs. Using bioinformatic data analysis and multiple biochemical tools, they also discovered that hsa_circ_001783 is necessary for BC progression and metastatic spread, mechanistically sponging miR-200c-3p. Circ_0005230 is another candidate that has a significant increase in expression when comparing both BC tissues and cell lines with normal tissue and cells, respectively (Xu Y. et al., 2018). Some clinical characteristics were taken into consideration, showing that patients with raised expression of circ_0005230 have lower 5-year OS rates. Also functioning as a miRNA sponge, this circRNA reduces miR-618 expression, indirectly elevating the expression of CBX8.

In some circumstances, circRNAs can even induce the spreading of tumors to adjacent organs such as hsa_circ_0052112 (Zhang H.D. et al., 2018). Functioning as an oncogenic circRNA, hsa_circ_0052112 enhances tumor cells to invade and migrate by sponging miR-125a-5p that acts as a tumor suppressor, inhibiting BAP1 oncogene. Similar to hsa_circ_0052112, hsa_circ_0007534 shows an inverse expression with miR-593 (Song and Xiao, 2018). The tumor suppressive role of this circRNA was confirmed both through overexpression and knockdown experiments, which proved that hsa_circ_0007534 can function as an oncogenic regulator in BC.

Knowing that the high expression of circ-Foxo3, Foxo3, and Foxo3 pseudogene decreases cell viability, Yang et al. planned to study their roles in breast carcinoma development. They found that the mRNA of Foxo3, the circRNA of Foxo3 and Foxo3 pseudogene are regulated by eight miRNAs: miR-149^∗^, miR-136^∗^, miR-138, miR-22, miR-433, miR-3614-5p, miR-762, and miR-3622b-5p. Furthermore, they discovered that the ectopic expression of these three transcripts could decrease cancer cell proliferation and cancer cell survival and tumor growth, confirming their tumor suppressive role (Yang et al., 2016).

CircTADA2As were also confirmed to have a tumor suppressive role in triple negative breast cancer (TNBC). In a large cohort of BC patients, both circTADA2A-E6 and circTADA2A-E5/E6 were found to be significantly decreased, and their low levels were linked to short survival rates. Focusing on circTADA2A-E6, Xu et al. (2019) demonstrated that this circRNA suppresses cellular growth, invasion, migration, and colony formation. Furthermore, circTADA2A-E6 has been shown to complementarily bind to miR-203a-3p and inhibit its repressive function on SOCS3, inducing a less aggressive cancer.

On the contrary, Tang et al. (2017) identified hsa_circ_ 0001982 to be up-regulated in BC cell lines and tumors, using microarrays analysis. *In vitro* gene modulation studies showed that hsa_circ_0001982 inhibits BC cell growth, invasion, and induce cell death by sponging miR-143. Similarly, He et al. proved the oncogenic role of a circRNA called circGFRA1 in TNBC. While up-regulation of circGFRA1 is correlated with poor prognosis, its knockdown inhibits proliferation and promotes apoptosis in TNBC. To assess the mechanism behind the functionality of this circRNA, He et al. (2017) used luciferase reporter assay and concluded that circGFRA1 and GFRA1 directly bind to miR-34. Taken together, these findings suggest that circGFRA1 regulates GFRA1 expression through sequestering miR-34 and may function as a sponge, confirming its regulatory function in TNBC.

Also, in TNBC, Zeng et al. identified circANKS1B pro-metastasis effect. They first demonstrated that this circRNA is overexpressed in TNBC tumors and cell lines. Furthermore, they demonstrated that it induces epithelial-to-mesenchymal transition (EMT) promoting BC metastasis in a murine cancer model. Mechanistically, circANKS1B sponges two miRNAs: miR-152-3p and miR-148a-3p, indirectly up-regulating USF1. USF1, being a transcription factor, induces higher levels of TGF-β1, activating the pro-metastatic signaling pathway TGF-β1/Smad. In summary, these results confirm the pro-tumorigenic function of circANSK1B in BC (Zeng et al., 2018).

Another circRNA up-regulated in TNBC, circAGFG1, was shown to promote cell proliferation, mobility and invasion *in vitro*, oncogenesis, and distant metastasis *in vivo*. According to these results, the level of this circRNA correlates with pathological grade, clinical stage and poor prognosis of TNBC patient. Functional studies showed that circAGFG1 may block the function of miR-195-5p relieving its repressive effect on cyclin E1 (CCNE1) mRNA, confirming its oncogenic role (Yang R. et al., 2019).

### Cervical Cancer

Often associated with HPV16 and 18 infection, CC arise from the transitional zone between the cylindrical and the squamous epithelium; most of them are squamous cell carcinomas or adenocarcinomas (Bosch et al., 1995). CC can remain loco-regional for a long time with a cervical extension from close to close, while invasive cancers are defined with basal membrane crossing (Waggoner, 2003). As genetic and epigenetic variations can predispose to this malignant gynecological tumor (Cordeiro et al., 2018), understanding the underlying molecular mechanisms remains a necessity.

CircAMOTL1 is one of the best-characterized circRNAs in CC. CircAMOTL1 is up-regulated in CC tissues compared to healthy adjacent tissues, and its expression is especially high in metastatic tumor tissues. At a phenotypical level, circAMOTL1 induces tumor development both *in vitro* and *in vivo*. Three databases, PITA, miRmap, and microT, suggested 61 potential binding miRNAs of circAMOTL1. MiR-485-5p, a binding candidate, was found significantly down-regulated in tumor tissues, therefore suggesting that circAMOTL1 enhances oncogenic activities in CC via the circAMOTL1/miR-485-5p axis. Further experiments provided evidence that the up-regulation of circAMOTL1 induces the overexpression of its host gene, AMOTL1. With all of the supporting data, circAMOTL1 is believed to play an oncogenic role in CC (Ou et al., 2019).

Also, very well-characterized is the role of circE7 in CC. A newly published paper has described the role of this viral circRNA with coding potential. HPV16 circE7 back-splicing junction was detected and characterized using Northern blotting and inverse RT-PCR of HPV16-infected cell lines. The circRNA was shown to be essential for coding E7 oncoprotein, which induces tissue proliferation and invasion in CC *in vitro* and *in vivo*. Because HPV plays regulatory roles in transcriptional and post-transcriptional activities in response to the differentiation state of epithelial cells, this circRNA formation can affect how HPV coordinates infection and immune evasion (Zhao J. et al., 2019). From this study, it is possible to further investigate the diagnostic and therapeutic implications of circE7 in CC.

Gao et al. (2017) elucidated the molecular basis of hsa_circ_0018289 on CC tumor formation. Among 45 up-regulated circRNAs detected by microarray, hsa_circ_00018289 was the one most significantly overexpressed in 35 CC tumors compared to the adjacent normal tissues. The loss-of-function experiments revealed its function in cancer cell proliferation and invasion. Via luciferase reporter assay, Gao et al. (2017) validated that hsa_circ_0018289 targets miR-497 and suppresses its expression. A circRNA isoform of eukaryotic translation initiation factor 4γ2 (circEIF4G2) was found to be up-regulated in CC tissues and was linked to unfavorable prognosis (Mao et al., 2019). *In vivo* and *in vitro* evidence highlighted the plausibility of circElF4G2 cancerous characteristics such as cell proliferation, colony formation, and metastasis. Mechanistically, this circRNA proved to inhibit miR-218, further influencing the downstream target of the miRNA, the transcription factor homeobox A1 (HOXA1). The increasing expression level of circEIF4G2 indirectly induces the expression of HOXA1 both at transcriptional and at translational levels. Although the axis of circEIF4G2/miR-218/HOXA1 has not been yet well elucidated, early findings have shown that HOMO genes family are associated with carcinogenesis and low survival rates in CC patients (Bitu et al., 2012; Eoh et al., 2017).

Xenopus kinesin-like protein 2 (TPX2) is another example of the many indirect targets of circRNAs via miRNAs. Jiang et al. (2014) previously reported an abnormal behavior of this microtubule-associated protein in CC progression and invasion via immunohistochemistry and RT-qPCR. Because miR-8075 can inhibit TPX2, the sponging effects of hsa_circRNA_101996 on miR-8075 indirectly generates more TPX2. This molecular mechanism was further suggested by Song et al. (2019) describing how the increased level of hsa_circRNA_101996 is associated with different stages of CC and induced proliferation, migration, and invasion.

After examining the characteristics and circRNA expression, Cai et al. (2019) confirmed the inducible patterns of hsa_circ_0000263 on tumor cell growth. This circRNA can affect post-transcriptional gene regulation, especially restraining the activity of miR-150-5p, indirectly regulating murine double minute 4 (MDM4) gene expression. By inhibiting miR-150-5p, this oncogenic circRNA eventually decreases the expression of p53 tumor suppressor, because MDM4 is a critical inhibitor of p53 (Wade et al., 2013). Hsa_circ_0067934 is another circRNA that displays tumorigenic properties in CC. In CC tissues, the expression of this circRNA is significantly up-regulated and is linked to lymphatic metastases (Hu et al., 2019). Knockdown experiments of hsa_circ_0067934 validated its capacity to induce tumor proliferation, colony formation, and EMT features. By providing luciferase assay data, Hu et al. (2019) showed that this circRNA mainly targets miR-545 and down-regulates its expression. This miRNA subsequently regulates the eukaryotic initiation factor 3C (EIF3C), which has been previously reported to suppress cell growth and induce cancer cells apoptosis (Hao et al., 2015; Zhao et al., 2017). Sometimes the dysregulated expression of a circRNA can affect more than one miRNA at the same time. The sponging effects were observed both for miR-518-5p and miR-519-5p by circRNA_8924 simultaneously, which induces the aggressive characteristics of CC tumors such as metastasis (Liu J.M. et al., 2018). Similarly, hsa_circ_0023404 is up-regulated in CC and restrains the activity of miR-136 (Zhang J.H. et al., 2018). Zhang J.H. et al. (2018) further knocked down this circRNA showing that it significantly suppresses proliferation, arrests cell-cycle progression, and inhibits cell migration and invasion in CC. *In vivo* research was used to understand the function of circRNA-000284, the authors reported that when decreasing the non-physiologically high levels of this circRNA, it prevents cells from proliferating and invading to adjacent organs. Moreover, if cirRNA-000284 expression is suppressed in CC cells, cell cycle arrest is promoted in G0/G1 phase and cancer growth is slowed down (Ma et al., 2018).

CircSMARCA5 was found to be down-regulated in CC while its high-levels slow-down CC cell growth, migration, invasion, and prompt cell cycle arrest *in vivo*. Furthermore, circSMARCA5 binds to miR-620 and significantly down regulates its expression. Tian et al. have demonstrated that this circSMARCA5/miR-620 regulatory axis leads to a suppression of invasion and proliferation confirming its involvement in CC (Tian and Liang, 2018).

Conversely, circ-ATP8A2 is up-regulated in CC tumors and cell lines, suggesting an oncogenic role. According to this, the down-regulation of circ-ATP8A2 inhibits cell growth, migration, and invasion and increases apoptosis, while overexpression circ-ATP8A2 results in a reverse phenotype. Mechanistically, circ-ATP8A2 blocks the function of miR-433, indirectly derepressing epidermal growth factor receptor (EGFR) mRNA (Ding and Zhang, 2019).

### Ovarian Cancer

Ovarian cancer is one of the most lethal gynecological cancers with very low survival rates because of its deep localization, leading to late and non-specific symptoms (Holschneider and Berek, 2000). Numerous diagnoses are made at the peritoneal carcinomatosis stage where the volume of the primary tumor and its intraperitoneal extension are considerable, requiring surgical treatment and chemotherapy. Only women with a nil or minimal post-surgical tumor residue have a chance of prolonged survival (Rosen et al., 2009; Malvezzi et al., 2016). Hence, modern OC management has shifted toward developing potential biomarkers for early diagnosis, risk assessment, prediction of treatment success, and treatment toxicity (Yang W.L. et al., 2017; Zhu et al., 2019). Because OC is highly controlled by different genetic pathways with multiple molecular characteristics, it is the utmost importance to understand dysregulation in cancer cells using genome wide screening methods. Having a long half-life in body fluids and specificity in cancer, circRNAs are being actively explored as biomarkers for OC diagnosis (Memczak et al., 2015; Zhang et al., 2017).

One of the best-characterized circRNA in OC is CDR1as, which has a potential function in cisplatin chemoresistance. In this study, CDR1as was lowly expressed in cisplatin-resistant OC patient tissues. The function of CDR1as in the acquisition of cisplatin chemoresistance was more strongly validated thanks to *in vitro* and *in vivo* experiments. Bioinformatics prediction analysis by two different databases suggested miR-1270 as a molecular target of CDR1as. Consistently, this miRNA was highly expressed in cisplatin-sensitive cells, obviously opposite to the trend observed with CDR1as. Furthermore, miR-1270 targets a tumor suppressor gene, SCAI—by binding to the putative binding sites on its 3′ UTR, it decreases SCAI expression level in cisplatin-sensitive cells. Data were more promising when Zhao’s group also detected a low level of CDR1as in serum exosomes, which suggested using this circRNA as a stable tool for detecting cisplatin-resistant OC tumors (Zhao Z. et al., 2019).

In an early OC-associated circRNA publication that was released in 2016, scientists detected numerous circRNA isoforms in primary OC specimens and matched peritoneal carcinomatosis and metastasized lymph nodes (Ahmed et al., 2016). Some genes involved in important signaling pathways such as NF-kB, PI3k/AKT, and TGF-β were found to be expressed differentially between the linear and the matched circRNAs, using paired-end RNA-Seq libraries from primary ovarian tumors, matched peritoneum, and lymph node metastases. CircRNA and mRNA levels exhibit an opposite trend; for example, the mRNAs of NF-kB, PI3k/AKT, and TGF-β are usually up-regulated in metastatic tissues, while the corresponding circRNAs are down-regulated. This differential expression pattern opens a promising direction in using these circRNA forms as biomarkers for highly heterogenous cancer transcriptomes.

Recently, Teng et al. published a comprehensive analysis of 7,333 circRNAs related to OC regulatory activities, in which the expressions of 2,431 were noticeably increased, and those of 3,120 were significantly decreased (Teng et al., 2019). Among all validated circRNAs, circHIPK3 showed the most down-regulated signals based on the sequencing data and displayed various tumor suppressive functions. Specifically, depleting circHIPK3 in OC cell lines promotes cell growth and migration to adjacent tissues, as well as negatively regulates the programed cell death mechanism. In high-grade serous OC (SOC), the most common pathological subtype of OC, there are up to 710 differentially expressed circRNAs. CircRNA1656 is the most differentially expressed among all and highly associates with OC patients tumor stage (Gao et al., 2019). Hu et al. (2018) demonstrated that circ-ITCH sponges miR-145 increase the expression of RASA1 and therefore inhibit tumor progression both *in vitro* and *in vivo*. Functionally, the down-regulation of miR-145 gives rise to RASA1 protein expression, inducing tumor proliferation, invasion, and migration. As a miR-370 sponge, hsa_circ_0061140 promotes cell proliferation and metastasis in OC cell lines SKOV3 and A2780, subsequently decreasing FOXM1 expression (Chen Q.Z. et al., 2018). Via RNA fluorescence *in situ* hybridization (FISH) and luciferase reporter assays, Chen et al. were able to detect the up-regulation of hsa_circ_0061140 and how tumor growth is promoted through the expression of this circRNA.

Finally, circEPSTI1 was found remarkably up-regulated in OC. A series of experiments were performed by Xie J. et al. (2019) showing that circEPSTI1 regulates EPSTI1 levels and OC development by inhibiting miR-942. They showed that circEPSTI1 inhibition suppresses cancer cell growth and invasion capacity, and induces programed cell death in OC, confirming its oncogenic role (Xie J. et al., 2019).

### Endometrial Cancer

The number of patients with EC has increased recently, while the age of diagnosis is earlier than before (Moore and Brewer, 2017). A recent report of EC showed that in 2017, in the United States, there were 61,380 newly diagnosed cases and over 10,920 deaths^1^. EC is categorized into two subtypes: type 1 lesions, often low-grade and hormonal sensitive, are one of the most frequent and have an optimal prognosis, while the second type is rare but more aggressive and at risk of recurrence, even for early stage tumors (Amant et al., 2005). Due to the severe mortality of EC in recent years, scientists are trying to develop specific predictive biomarkers for endometrial malignancies. The diagnosis and treatment of ECs has recently been greatly improved thanks to advances in the knowledge of regulatory pathways involved in tumor initiation and progression. Precise molecular characterization of the disease led to the developing of targeted therapies and diagnostic tools for EC patients, such as PP2A, a tumor suppressive heterotrimeric protein phosphatase type 2A (Remmerie and Janssens, 2019). Preventing endometrial tumorigenesis and tumor invasion, PP2A has been shown to be altered in more than 40% of EC.

Compared to BC, OC, and CC, the function of circRNAs in EC is less characterized. The most characteristic study on circRNAs in EC was performed by Ye et al. (2019b). Using circRNA sequencing, the authors conducted a screening on the expression of 75,928 different circRNAs in grade 3 EC tumors versus adjacent healthy endometrial tissues. Among all, 25,735 circRNAs were overexpressed and 36,432 were down-regulated. They also ranked the top five circRNAs that have the highest expression and lowest expression in EC tissues. Hsa_circ_0039569, hsa_circ_0001523, hsa_circ_0001610, hsa_circ_0001400, and hsa_circ_0007905 were substantially overexpressed, while hsa_circ_0000437, hsa_circ_0001776, hsa_circ_0009043, hsa_circ_0000471, and hsa_circ_0014606 were the most down-regulated in their analysis. Further bioinformatic analysis demonstrated that circRNAs and miRNAs build a complex regulatory network; deregulated circRNAs potentially sponge 451 miRNAs. Based on this regulatory network, the hsa-circ-0039659/hsa-miR-542-3p/hsa-let-7c-5p pathway has been suggested as the most important one in predicting grade 3 EC (Ye et al., 2019b).

Another circRNA has been recently revealed to play a role in the tumorigenesis of EC: circ-ZNF91 (Chen B.J. et al., 2018). Circ-ZNF91 functions as a miRNA sponge, inhibiting the expression of miR-23b and miR-199. Moreover, Chen B.J. et al. (2018) performed a global expression profile of circRNAs to compare cancer and healthy endometrial tissues. Based on their findings, the ratio of circRNAs to linear RNA isoforms was lower in EC than in healthy endometrial tissues, 23.9 and 30.1%, respectively. There were up to 120 circRNAs differentially expressed in EC tissues, out of which HSPG2 and RP11-255H23.4, two ciRNAs, were found to be expressed only in healthy tissues. Surprisingly, their mRNA isoforms increased significantly in EC tissues. Although this study did not establish further details on how each individual circRNA expression leads to tumor initiation and progression, it provided a foundation for future investigations on EC-associated circRNA functions and mechanisms.

## circRNAs as Potential Circulating Biomarkers and Therapeutic Targets

CircRNAs are potential non-invasive biomarkers. In BC, Yin et al. (2018) investigated plasma circRNAs’ expression with the aim of discovering valuable diagnostic biomarkers. They identified hsa_circ_0001785 as a stable biomarker for the diagnosis and progression of BC. Data showed that this circRNA has an AUC of 0.784, and if combined with two other established biomarkers, carcinoembryonic antigen (CEA) and carcinoma antigen 15-3 (CA 15-3), the AUC increases to 0.839 (Yin et al., 2018).

In CC, Li et al. (2019) developed a new microarray capable of profiling circRNAs. Interestingly, the newly developed tool seemed to be superior to RNA-seq and the authors also tested it when profiling the circRNAs from plasma of patients with CC. It was possible to detect around 18,000 circRNAs in the plasma of CC patients and the expression of 2,787 circRNAs was deregulated after surgery for tumor removal. The diagnostic and prognostic power of circRNAs was not further tested, but it is plausible to speculate that they can be used as non-invasive biomarkers for CC (Li et al., 2019).

Knowing that serum circSETDB1 is a tumor-promoting circRNA and is up-regulated in SOC, Wang W. et al. (2019) chose to investigate the potential role of this circRNA as a biomarker. First, they assessed the capacity of this circRNA to separate SOC patients from healthy controls. A ROC curve analysis showed that serum circSETDB1 expression can be used to discriminate SOC patients from healthy controls, with an AUC of 0.8031 and a sensitivity of 78.33% and specificity of 73.33%. The same circulating circRNA was used to separate primary chemoresistant SOC patients from primary chemosensitive ones. Data showed that circSETDB1 can be used to diagnose chemosensitivity with an AUC of 0.8107 and a specificity of 76.74% and sensitivity of 77.78%. Finally, Wang W. et al. (2019) investigated if serum circSETDB1 levels can be used as a predictive tool for progression free survival (PFS). Patients with low levels of circSETDB1 had a mean PFS of 18.9 months and patients with high levels had a mean PFS of 13.2 months (Log Rank = 6.815, *P* = 0.006). Taken together, these data prove that circSETDB1 is a promising non-invasive diagnostic and prognostic biomarker for SOC (Wang W. et al., 2019). Serum circMAN1A2 was found up-regulated in several cancers including OC, which was investigated as a potential biomarker by Fan et al. Even if data appear less promising in OC than in other malignancies, circMAN1A2 still has an AUC of 0.694, a sensitivity of 0.583, and a specificity of 0.806. According to the authors, further studies are necessary to confirm or infirm the potential role of this circRNA as a biomarker for OC (Fan et al., 2019).

In EC, Xu H. et al. (2018) conducted a pilot study and discovered that serum circulating extracellular vesicles from EC patients contain 209 up- and 66 down-regulated circRNAs compared to matched healthy volunteers. The authors further validated the up-regulation of has-circ_01090406 and has_circ_0002577, but did not test the diagnostic power of these circular transcripts.

The CircRNAs described above represent only a fraction of the promising diagnostic and prognostic potential of these molecules in BC and female reproductive system cancers. However, it is important to note that further studies will be required before these circRNAs can be used in further clinical settings as diagnostic or prognostic biomarkers.

Similar to coding genes or well-studied non-coding genes (i.e., miRNAs), circRNAs can be classified as tumor suppressors and oncogenes. Therefore, we can envision two different therapeutic approaches: inhibiting the circRNAs, which are carcinogenic and overexpressed in tumor tissues, or restoring circRNAs with tumor suppressor functions, which are down-regulated in tumors.

Tumor suppressor circRNAs were reported to act as potent endogenous sponges that bind oncogenic miRNAs (oncomiRs) and inhibit their function (Dragomir and Calin, 2018a). Artificially synthesized tumor suppressor circRNAs are promising anti-miRNA therapy. Because it is known that not only one type of miRNA is overexpressed in specific cancer type, these artificial constructs can be designed to bind and inhibit multiple oncomiRs simultaneously. Additionally, artificial circRNAs can have multiple binding sites for the same miRNA, similar to the well-known sponge of miR-7, that harbors over 70 binding regions for this miRNA (Hansen et al., 2013; Memczak et al., 2013). Hence, circRNAs seem to be the ideal inhibitors for oncomiRs. Recently, synthetic circRNA based-therapy for gastric and esophageal cancers was developed and proved to be efficient (Liu X. et al., 2018; Wang Z. et al., 2019), and so similar strategies may also be used for BC and female reproductive system cancers. Moreover, it was also reported that endogenous circRNA have an anti-viral function at the cellular level (Tagawa et al., 2018). Artificial circRNAs could be designed to inhibit the replication of oncoviruses and to design preventive therapies for cancers caused by viral infections (i.e., CC).

There are some clear advantages of circRNA restoration therapies compared to other RNA therapies: circRNAs have a longer half-life compared to mRNAs, so the dose can be reduced and the administration of the treatment can be infrequent. There are also possible disadvantages. For example, it is not yet clear if artificial circRNAs can activate the immune system, like miRNAs, and induce systemic inflammatory response syndrome (SIRS), one of the most frightening adverse events of RNA therapy. Because of the similar structure to some viral particles [the first described circRNAs were viroids (Sanger et al., 1976)], this approach could be very dangerous.

Oncogenic circRNAs are overexpressed and usually inactivate tumor suppressor miRNAs. Inhibiting oncogenic circRNAs has not yet been explored, but we can imagine several strategies. First, it is possible to block the biogenesis of circRNAs using small molecules similar to the therapies developed for miRNAs. Second, we can envision complementary, small RNA molecules that bind the sites of the circRNA responsible for sponging and inhibiting tumor suppressor miRNAs. It is important to mention that this single strand RNA molecule needs to have a higher affinity for the circRNAs which target miRNAs. This therapy is similar to miRNA mask therapy. Finally, it is possible to directly induce the degradation of circRNAs using RNA inference. One possible solution is short interfering RNAs (siRNAs), which can be further chemically modified to increase efficiency (i.e., locked nuclei acids) (Shah et al., 2016; Petrescu et al., 2019). It is important to mention that, in order to induce the knockdown of circRNAs, siRNAs need to target the back-splice junction of the circular transcript (Kristensen et al., 2019).

## Studying circRNAs Via Database

To make research more accessible, it is urgent to create comprehensive databases for circRNAs and their related diseases and/or targets to be able to generate more in-depth analyses. In the past decade, using next-generation sequencing and bioinformatics, various circRNA studies were collected and integrated into circRNA databases. Two well-known circRNA datasets, circBase, and circFunBase, provide general information on each circRNA expression (Glazar et al., 2014; Meng et al., 2019). These data collections consist of all circRNA-related research from 2013 until the present as well as over 150,000 circRNA genomes sequences from diverse species. More specifically, some databases introduce visualized circRNA–miRNA interaction networks to create a detailed platform of circRNA–disease relationships such as Circ2Traits (Ghosal et al., 2013). CircNet is one of the most widely used databases, gathering the genomic annotation and sequence of circRNA isoforms, tissue-specific expression levels, and miRNA or gene-related interactome maps (Liu et al., 2016). An example of a more exhaustive database, CSCD (cancer-specific circRNA database), is derived from a total of 228 RNA or polyA(-) RNA-seq samples from malignant and normal *in vitro* models. This powerful dataset contains 272,152 cancer-specific circRNAs in different types of tumors (Xia et al., 2018). MiOncoCirc is another dataset of circRNAs that represents all circRNAs regulating primary tumors, metastasis, and even rare cancers (Vo et al., 2019). There are other circRNA databases that have been used in the past decade to detect and validate targets in numerous studies related to this new member of the ncRNA family such as CircRNADb, CircInteractome, CIRCpedia v2, and TCSD (Chen et al., 2016; Dudekula et al., 2016; Zheng L.L. et al., 2016; Aghaee-Bakhtiari, 2018; Dong et al., 2018). A more user-friendly platform is Circ2Disease, which can be used to search how many circRNAs are deregulated in various diseases including malignant tumors. This database provides thorough information about the expression patterns of circRNAs, the experimental technique of detection, and literature reference on PubMed (Fan et al., 2018).

We have summarized 11 circular RNA databases in Table 2. However, most of the current databases have not been updated since 2017–2018, while, in the field of breast and gynecological cancers, most circRNA-related discoveries have been made in late 2018 and 2019. Presently, over 700 scientific papers have been published investigating the association of circRNAs in regulating malignant pathways, but we still lack a thorough and complete perspective on how different circRNAs function and how they are regulated. We believe it is of great significance to update the databases more frequently and develop their systems to be more generic, organized, and viable. It will make it easier for scientists to get access and link the rules of circRNA biology and reveal their functions in studying cancer.

**TABLE 2:** CircRNAs databases.

**Name**	**Website**	**Description**	**References**
CircInteractome	http://circinteractome.nia.nih.gov/	Predicts and maps the binding sites for RBPs and miRNAs on reported circRNAs.	Dudekula et al., 2016
CircBase	http://www.circbase.org/	Public circRNA datasets and custom python scripts to discover circRNA.	Glazar et al., 2014
CircFunBase	http://bis.zju.edu.cn/CircFunBase/	Utilizes 7,000 manually curated functional circRNA entries.	Meng et al., 2019
Circ2trait	http://gyanxet-beta.com/circdb/index.php	CircRNAs and their related diseases regulation.	Ghosal et al., 2013
CircNet	http://circnet.mbc.nctu.edu.tw/	Utilizes transcriptome sequencing datasets from circRNA expression in 464 RNA-seq samples.	Liu et al., 2016
CSCD	http://gb.whu.edu.cn/CSCD/#	Cancer-specific circRNA database.	Xia et al., 2018
MiOncoCirc	https://mioncocirc.github.io/	Cancer circRNA database constructed from clinical cancer samples.	Vo et al., 2019
CIRCpedia v2	http://www.picb.ac.cn/rnomics/circpedia/	CircRNA annotations retrieved from 180 RNA-seq datasets in six different species.	Dong et al., 2018
TCSD	http://gb.whu.edu.cn/TSCD	Tissue-specific circRNA database.	Aghaee-Bakhtiari, 2018
CircRNADb	http://reprod.njmu.edu.cn/circrnadb	circRNA database annotated (in particular in humans.)	Chen et al., 2016
Circ2Disease	http://bioinfo.snnu.edu.cn/CircR2Disease/	739 manually curated circRNA entries associated with 100 different diseases.	Fan et al., 2018

## Conclusion

CircRNAs are belived to be involved in the tumorigenesis of different types of malignancies, including female reproductive system and breast cancers, and similar to other ncRNAs (Jin et al., 2013; Zhou et al., 2014), they have prognostic, diagnostic, and therapeutic potential. The high conservation between species, the specific expression, and the variety of their roles suggest that circRNAs are specifically involved in physiological cellular mechanisms (Ebbesen et al., 2017). As demonstrated in this review, the dysregulation in gene expression of circRNAs is believed to be one of the major mechanisms leading to the development and progression of gynecological cancers. In the blossoming era of exploiting genetic determinants in cancer biology, the significance of circRNAs is beginning to be recognized and their functionalities are beginning to be elucidated. There is copious evidence indicating that circRNAs will play a tremendous role in the future care of cancer patients via diagnostic and therapeutic approaches. If the footprints of circRNAs are yet to be confirmed, circular transcripts might be a major, ground-breaking target for therapies: either to block overexpressed, pro-tumorigenic circRNAs or to restore down-regulated tumor suppressive circRNAs. With the assistance of a growing number of circRNA databases available, scientists will have access to established studies and can further develop their research directions.

## Author Contributions

AT, GMC, MD, and GAC: conception and design. CI, MD, and GAC: provision of study materials. AT, GMC, LB, MC, and MD: collection and assembly of data. AT, GMC, LB, PR, SJ, EG, MD, and GAC: manuscript writing. MC and MD: figure design. All authors gave the final approval of the manuscript.

## Conflict of Interest

The authors declare that the research was conducted in the absence of any commercial or financial relationships that could be construed as a potential conflict of interest.

## Footnotes

1 https://www.cancer.org/research/cancer-facts-statistics/all-cancer-facts-figures/cancer-facts-figures-2017.html
